# MRPL47 deficiency drives mitochondrial dysfunction *via* ROS-p38-p21 signaling in non-small cell lung cancer

**DOI:** 10.1016/j.jbc.2025.111058

**Published:** 2025-12-15

**Authors:** Nikita Bhandari, Yengkhom Ghanapriya Devi, Disha Acharya, Vinita Bhat, Annesha Chatterjee, Shweta Yalshetti, Sharathchandra Arandkar, Bal Krishna Chaube, Sudhanshu Shukla

**Affiliations:** 1 Department of Biosciences and Bioengineering, Indian Institute of Technology Dharwad, Dharwad, India; 2 Advanced Centre for Treatment, Research and Education in Cancer (ACTREC), Tata Memorial Centre, Navi Mumbai, India

**Keywords:** CDKN1A (cyclin-Dependent Kinase inhibitor 1A), cellular senescence, mitoribosome, mitogen-activated protein kinase (MAPK), oxidative phosphorylation (OXPHOS), reactive oxygen species (ROS)

## Abstract

Mitoribosomes are pivotal for cellular energy metabolism through the synthesis of proteins essential for the oxidative phosphorylation system. Although mitoribosomal dysregulation has been implicated in cancer, the genomic landscape of mitoribosomal proteins (MRPs) in nonsmall cell lung cancer (NSCLC) remains largely uncharacterized. In this study, we conducted a comprehensive analysis of expression, copy number variations, and mutations of MRPs using data from TCGA-NSCLC patients. This screen identified MRPL47 as a significantly amplified and overexpressed mitoribosomal gene in NSCLC. Validation across three independent datasets (n = 1513) confirmed MRPL47 as a robust and independent prognostic marker for poor survival. Functionally, MRPL47 inhibition significantly reduced NSCLC cell proliferation and migration. Intriguingly, MRPL47 depletion selectively impaired the translation of a subset of mitochondrial proteins, rather than causing a global defect, leading to impaired assembly of electron transport chain Complexes I and III. This resulted in a defective oxidative phosphorylation system, characterized by decreased ATP synthesis and elevated mitochondrial reactive oxygen species (ROS) levels. Transcriptomic analysis revealed a significant downregulation of E2F pathway activity in MRPL47-knockdown cells, with MRPL47 expression correlating with E2F target gene expression at both RNA and protein levels. Mechanistically, MRPL47 knockdown induced ROS accumulation, which promoted p38 phosphorylation and subsequent upregulation of p21. Increased p21, in turn, led to Rb hypophosphorylation, thereby inhibiting E2F activity and inducing G1 cell cycle arrest and senescence. Altogether, these findings establish that MRPL47 is amplified and overexpressed in NSCLC, functions as a strong prognostic predictor, and critically promotes tumor progression by modulating mitochondrial function and the ROS-p38-p21-Rb-E2F signaling axis.

Non-Small Cell Lung Cancer (NSCLC) is the most common form of lung cancer, constituting over 80% of lung cancer occurrences and serving as the most common cause of cancer-related mortality worldwide (1, 2, 3). The discovery of novel mechanisms and biomarkers is essential for advancing NSCLC management. These findings enable early diagnosis, guide personalized therapies, and address treatment resistance. By uncovering new molecular pathways and therapeutic targets, we can develop tailored treatments for NSCLC's heterogeneity. Novel biomarkers improve prognostic accuracy, predict treatment response, and monitor therapy efficacy in real time. Such advancements improve patient outcomes, optimize clinical decision-making, and reduce healthcare costs.

Mitochondria are essential organelles for cellular energy generation through oxidative phosphorylation. Mitoribosomes facilitate the production of proteins encoded by mitochondrial DNA that are crucial for the function of oxidative phosphorylation (OXPHOS) (4). Dysregulation of mitoribosomes promotes cancer by altering mitochondrial translation and apoptosis regulation (5). Conversely, perturbations in oncogenic signaling pathways can impact cellular metabolism and hence influence mitochondrial activity (6). Genetic and epigenetic modifications among cancer cells often promote a shift in metabolic pathways, switching from mitochondrial respiration to anaerobic glycolysis (Warburg effect) (7). This transition towards the Warburg effect may be attributed to mitochondrial dysfunction along with alterations in oxidative phosphorylation activity (8). Delineating these genetic abnormalities can boost the clinical outcomes by uncovering novel therapeutic targets, early detection biomarkers, and enhanced prognostic indicators.

The mitoribosome, a mitochondrial-specific ribosome, synthesizes core subunits of the OXPHOS complexes essential for cellular energy production. Structurally distinct from cytoplasmic ribosomes, it is membrane-tethered and enriched with mitochondrion-specific proteins (MRPs) that enable co-translational insertion of hydrophobic OXPHOS subunits into the inner mitochondrial membrane (9). Dysregulation of mitoribosomal function has been increasingly linked to cancer, where altered mitochondrial metabolism contributes to tumorigenesis. For example, a pan-cancer study identified alterations in 20 MRPs associated with the early stages of tumor formation (10). Additionally, 40 MRPs were found to be upregulated in breast cancer compared to normal tissues. Notably, MRPL52, a component of the large subunit of the mitoribosome, is a transcriptional target of HIF1α and is overexpressed in breast cancer relative to adjacent normal tissue (11). Mechanistically, MRPL52 helps breast cancer cells resist hypoxia-induced apoptosis. Furthermore, MRPL13 is regulated by lactate levels; its loss leads to defects in oxidative phosphorylation (12). MRPL33 is regulated at the splicing level by hnRNPK, and inhibition of its large variant results in reactive oxygen species (ROS) accumulation (13). MRPS23 serves as a crucial regulator of cell proliferation across various cancers, including breast cancer (14). Similarly, MRPL35 is upregulated in colorectal cancer and plays a role in regulating growth and apoptosis (15). These findings underscore the critical involvement of MRPs in the development and progression of multiple cancer types.

The OXPHOS complexes in mitochondria are crucial sources of reactive oxygen species (ROS), with approximately 90% of cellular ROS generated during this process. Complex I is a significant contributor to ROS production, primarily generating superoxide in the mitochondrial matrix, while Complex III is identified as the major site for ROS generation through the Q cycle (16). Complex II also produces ROS, albeit to a lesser extent (17). While low levels of mitochondrial ROS play important roles in cellular signaling and metabolic adaptation, excessive ROS production can lead to oxidative damage and is implicated in various diseases, including cancer (18). Understanding the dynamics of ROS production at different OXPHOS complexes is essential for exploring their roles in cancer progression and potential therapeutic strategies targeting oxidative stress-related pathologies.

This study explores the role of MRPL47 in cancer cells, emphasizing its influence on mitochondrial function and tumor progression. We analyzed expression, copy number variations, and mutation data from The Cancer Genome Atlas (TCGA) in NSCLC to delineate the regulatory landscape of. Our findings reveal that MRPL47 is frequently amplified and overexpressed in NSCLC. Knockdown of MRPL47 using shRNA significantly impaired cancer cell proliferation, migration, and colony formation. We demonstrated that MRPL47 selectively regulates the mitochondrial translation of specific OXPHOS proteins rather than affecting all mitochondrial proteins, leading to reduced enzymatic activity in electron transport chain complexes I and III, increased mitochondrial ROS levels, and a decrease in mitochondrial membrane potential (ΔΨM). Further investigations uncovered that MRPL47 modulates mitochondrial ROS signaling through the p38 -p21-pRB pathway. Collectively, these findings position MRPL47 as a critical regulator of mitochondrial dynamics and ROS balance in tumor progression, suggesting new therapeutic strategies targeting this pathway.

## Results

### Genomic alterations in MRPL47 reveal its oncogenic potential in NSCLC

**Figure 1:**
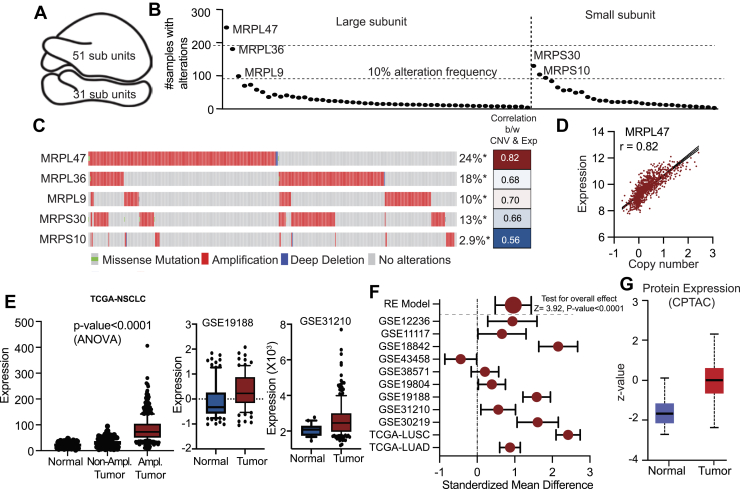
**Amplification and Overexpression of MRPL47 contributes to reduced overall survival in non-small cell lung cancer (NSCLC).***A,* structural organization of a human mitoribosome consisting of a lager and a smaller subunit, essential for mitochondrial translation. *B,* the genomic analysis of top five MRP genes across NSCLC samples exhibiting an alteration frequency >10%, with MRPL47 displaying the highest alteration rate. *C,* the cBioPortal analysis highlights distinct genetic alterations in candidate MRP genes among NSCLC patients, with the frequency of gene alterations represented as percentages. MRPL47 exhibited the highest alteration frequency (24%), primarily driven by amplifications. *D,* graph depicting a positive correlation between copy number variations and gene expression for the five identified MRP genes, with MRPL47 exhibiting the strongest correlation (r = 0.82). *E,* MRPL47 expression levels categorized by copy number variation status in LUAD and LUSC subtypes. Amplified (Amp.) samples displayed.significantly higher expression levels than normal (Nor.) or diploid (Dip.) samples (*p* < 0.0001). *F,* immunohistochemistry analysis of MRPL47 expression in lung cancer and normal tissue. Lung cancer samples exhibit stronger MRPL47 staining (*bottom panel*) compared to normal lung tissue (*top panel*), indicating increased MRPL47 protein expression in tumor cells.*G,* bar graph indicating the distribution of MRPL47 expression levels across normal and lung cancer samples. Lung cancer samples predominantly exhibit medium to high MRPL47 expression, whereas normal samples show lower or undetectable expression, suggesting MRPL47 upregulation in lung cancer. LUAD, lung adenocarcinoma; LUSC, lung squamous cell carcinoma.

The 55S mammalian mitoribosome is a complex molecular machine uniquely adapted for mitochondrial translation. It is composed of two distinct subunits, a large 39S and a small 28S subunit, and includes 82 nuclear-encoded mitochondrial ribosomal proteins (MRPs) in addition to mitochondrial-encoded RNA components (Fig. 1*A*) (19, 20). To investigate the prevalence of genetic aberrations in nuclear-encoded MRP genes within NSCLC, we analyzed genomic and transcriptomic data sourced from TCGA. Our analysis identified five MRP genes—MRPL47, MRPL36, MRPL9, MRPS30, and MRPS10—that exhibited a high frequency of genomic alterations, defined as occurring in >10% of samples. Among these, MRPL47 showed the highest alteration frequency (Fig. 1*B*). Importantly, no recurrent mutations were detected in these frequently altered MRP genes, suggesting that the functional integrity of the wild-type proteins is critical for NSCLC development and/or progression. Utilizing the cBioPortal for Cancer Genomics, we determined that all five of these genes were significantly amplified in NSCLC patient samples, and these amplifications correlated strongly with increased gene expression levels (Fig. 1*C*). MRPL47, being the most amplified MRP gene, showed a robust linear correlation between expression and copy number variation (Fig. 1*D*). Consistent with this, we showed that copy number amplification drives MRPL47 overexpression in lung cancer, with amplified samples exhibiting significantly elevated MRPL47 expression compared to normal and diploid samples (*p*-value< 0.0001) (Fig. 1*E*). Supporting these findings, two independent microarray datasets (GSE19188 and GSE31210) demonstrated significantly increased MRPL47 expression in NSCLC samples (Fig. 1, *F* and *G*). Furthermore, analysis using the Lung Cancer Explorer database confirmed significantly elevated MRPL47 expression in NSCLC compared to normal lung tissues (Fig. 1*H*). At the protein level, data from the clinical proteomic tumor analysis consortium dataset revealed increased MRPL47 protein abundance in NSCLC samples relative to corresponding normal samples (Fig. 1*I*). Analysis using the University of California, Santa Cruz genome browser indicated that MRPL47 is a highly conserved gene with significant promoter activity (Fig. S1*A*). Cumulatively, these data indicate that the observed overexpression of MRPL47 in NSCLC is largely driven by recurrent genetic amplification. Extending our analysis, we examined MRPL47 expression and amplification across a panel of other cancer types, finding consistent amplification and overexpression in all types investigated (Fig. S1, *B*–*D*). MRPL47 expression also demonstrated high specificity and sensitivity in distinguishing cancerous from normal tissues across these diverse datasets (Fig. S1*E*).

### MRPL47 as a robust prognostic marker in NSCLC

**Figure 2:**
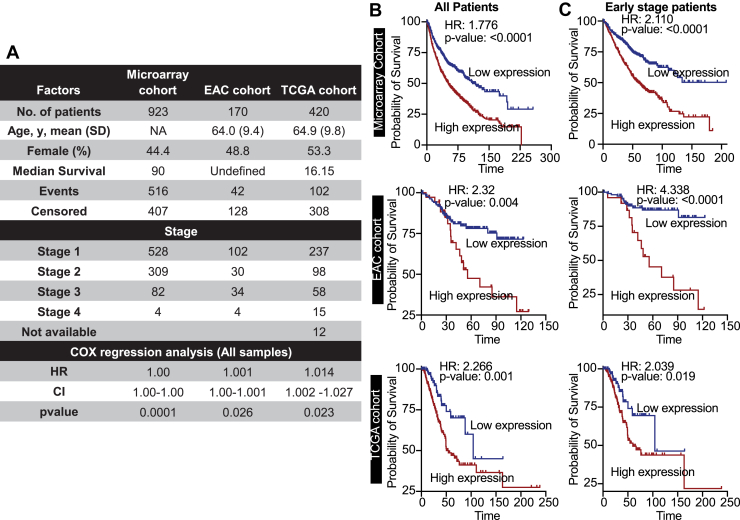
**MRPL47 is a strong prognostic marker for NSCLC:***A,* the table detailing the clinicopathological features of different cohort of patient's data utilized in this study. The table also shows the results of Cox regression analysis of the MRPL47. *B,* the Kaplan-Meier plots showing the survival of NSCLC patients divided based on MRPL47 expression. The cohort names are mentioned along with the plot. *C,* the Kaplan-Meier plot showing the survival of early stage NSCLC patients in given cohorts. Patients were divided into two groups based on MRPL47 expression. NSCLC, non-small cell lung cancer.

Given the observed overexpression of MRPL47 in NSCLC, we investigated its potential as a prognostic biomarker. This analysis utilized a combined total of 1513 NSCLC patients from three independent datasets: a microarray cohort from KM Plotter (n = 923), an East Asian cohort (EAC; n = 170), and TCGA cohort (n = 420). Detailed clinicopathological characteristics of these cohorts are presented in Figure 2*A*.

Univariate Cox regression analysis initially established that elevated MRPL47 expression was a significant predictor of poorer patient survival across all three cohorts (Fig. 2*A*). To further explore this, patients were dichotomized based on median MRPL47 expression (MRPL47-high *versus* MRPL47-low), and Kaplan-Meier survival analysis was performed. Consistent with the Cox regression findings, high MRPL47 expression was significantly associated with inferior overall survival in the Microarray Cohort (Hazard Ratio [HR] = 1.77, *p* < 0.0001), the EAC cohort (HR = 2.32, *p* = 0.004), and the TCGA Cohort (HR = 2.266, *p* = 0.001) (Fig. 2*B*).

In advanced stages of NSCLC, the disease has typically undergone significant locoregional spread or distant metastasis. Consequently, therapeutic objectives often transition from curative intent to focus on disease control, palliation of symptoms, and prolongation of progression-free or overall survival. While prognostic biomarkers retain relevance in advanced NSCLC, their principal impact in guiding adjuvant treatments with curative potential is most pronounced in early-stage disease. Therefore, we specifically assessed the prognostic significance of MRPL47 expression in early-stage (Stage I and Stage II) NSCLC. Patients within these stages were similarly stratified into MRPL47-high and MRPL47-low expression groups. In this subgroup analysis, high MRPL47 expression remained significantly associated with poorer survival in the Microarray Cohort (HR = 2.11, *p* < 0.0001), the EAC cohort (HR = 4.338, *p* < 0.0001) and TCGA cohort (HR = 2.039, *p* = 0.019) (Fig. 2*C*).

Furthermore, an association between MRPL47 copy number alterations and adverse survival outcomes was also identified (Fig. S1*F*). Notably, MRPL47 expression demonstrated prognostic significance, predicting poor survival in various other cancer types as well (Fig. S1*G*). Collectively, these findings across multiple independent cohorts consistently demonstrate that elevated MRPL47 expression is strongly associated with unfavorable overall survival in NSCLC patients, including those with early-stage disease.

### MRPL47 is required for the cell proliferation and migration

**Figure 3:**
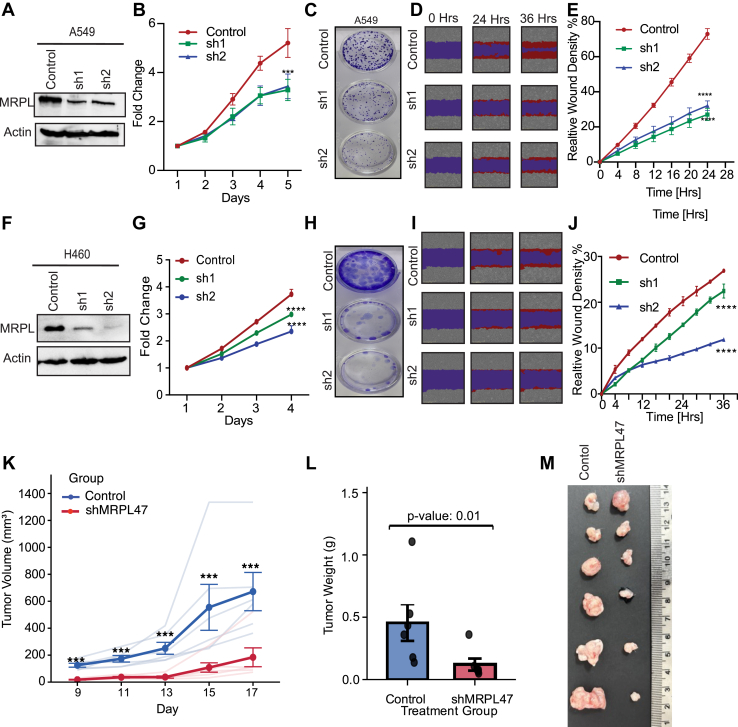
**MRPL47 knockdown suppresses cell proliferation, colony formation, migration an in-vivo tumor growth LUAD cell lines.***A* and *F,* Western blot analysis confirming MRPL47 knockdown using two independent shRNAs in A549 (*A*, Same blots are used in Fig. S2*I*), H460 (*F*) cells. *B and G,* cell proliferation assay demonstrating reduced proliferation in MRPL47 silenced cells compared to control in A549 (*B*) and H460 (*G*) cells over a given period. *C and H,* colony formation assay showing decreased colony-forming ability in MRPL47 knockdown cells in A549 (*C*), H460 (*H*) cell lines. *D and I* representative images of Scratch wound-healing assay displaying delayed wound closure in MRPL47 silenced cells compared to controls in A549 (*D*) and H460 (*I*) cells *E and J* quantification of wound-healing assay indicating significantly reduced migration capacity in MRPL47 depleted cells in A549 (*E*) and H460 (*J*) cells over a time period of 24 h. *K,* control and shMRPL47 H460 cells were injected in animal flank and tumor volume was measured and plotted at given time points. *L,* control and shMRPL47 H460 cells were injected in animal flank and tumor weight was measured and plotted after sacrificing animals. *M,* control and shMRPL47 H460 cells were injected in animal flank and tumors were removed and shown after sacrificing animals. *Statistical significance:* (∗∗∗denotes *p* < 0.001; ∗∗∗∗denotes *p* < 0.0001). Error bars represent mean ± SD from triplicate experiments.

To investigate the impact of MRPL47 on cellular growth, three NSCLC cell lines, A549, H460 and H1299, were selected for the experiments. Two distinct shRNAs targeting MRPL47 RNA were used for knockdown, which was subsequently validated by analyzing the protein expression of A549 cells (Fig. 3*A*). Interestingly, we observed a decrease in the proliferation of A549 MRPL47 knockdown (Fig. 3*B*). Colony suppression assay also showed that MRPL47 knockdown cells show reduced long-term cell growth in A549 cell line (Fig. 3*C*). Next, we performed a wound-healing assay displaying a reduced wound healing as compared to the control cells (Fig. 3*D*). These observations were replicated in H460 and H1299 cells (Figs. 3, *F*–*J* and S2, *A*–*E*). We also measure the effect of MRPL knockdown on apoptosis using Annexin V-PI staining. We observer increased Annexin V-PI staining in MRPL47 knockdown cells compared to control cells (Fig. S2, *F*–*H*). This observation was further confirmed using Western blot for Caspase three levels (Fig. S2*I*). In conclusion, MRPL47 is essential for cancer cell growth and proliferation through its role in mitochondrial function. Silencing of MRPL47 in lung adenocarcinoma (LUAD) cells inhibited proliferation, growth, and migration, highlighting its potential as a therapeutic target.

### MRPL47 is required for the tumor cell growth

To validate our *in vitro* findings and evaluate the therapeutic potential of MRPL47 inhibition, we performed xenograft experiments in nude mice. One million H460 cells (either control or MRPL47 knockdown) were subcutaneously injected into the flanks of nude mice (n = 6 per group). Tumor growth was monitored at regular intervals on days 9, 11, 13, 15, and 17 post-injections by measuring tumor dimensions using digital calipers. We observed a significant reduction in tumor growth in the MRPL47 knockdown group compared to the control group. As shown in Figure 3*K*, the tumor volume was markedly decreased in mice bearing MRPL47-depleted H460 cells throughout the observation period. The difference in tumor volume became increasingly pronounced over time, with statistical significance achieved by day 11 and maintained through day 17 (*p* < 0.05). By the endpoint of the experiment (day 17), the mean tumor volume in the MRPL47 knockdown group was ∼60% lower than that of the control group, demonstrating the critical role of MRPL47 in sustaining tumor growth *in vivo*. At the experimental endpoint, mice were sacrificed, and tumors were carefully excised and weighed. Consistent with the tumor volume measurements, the endpoint tumor weights in the MRPL47 knockdown group were significantly lower compared to the control group (Fig. 3*L*, *p* < 0.01). Representative images of the excised tumors from both groups are presented in Figure 3*M*, clearly demonstrating the visible difference in tumor size between control and MRPL47 knockdown xenografts. These *in vivo* findings corroborate our *in vitro* observations and establish that MRPL47 is essential for tumor growth and progression in a physiologically relevant animal model.

### MRPL47 is not a universal mitoribosome subunit; rather, it specifically regulates the translation of certain mitochondrially encoded proteins

**Figure 4:**
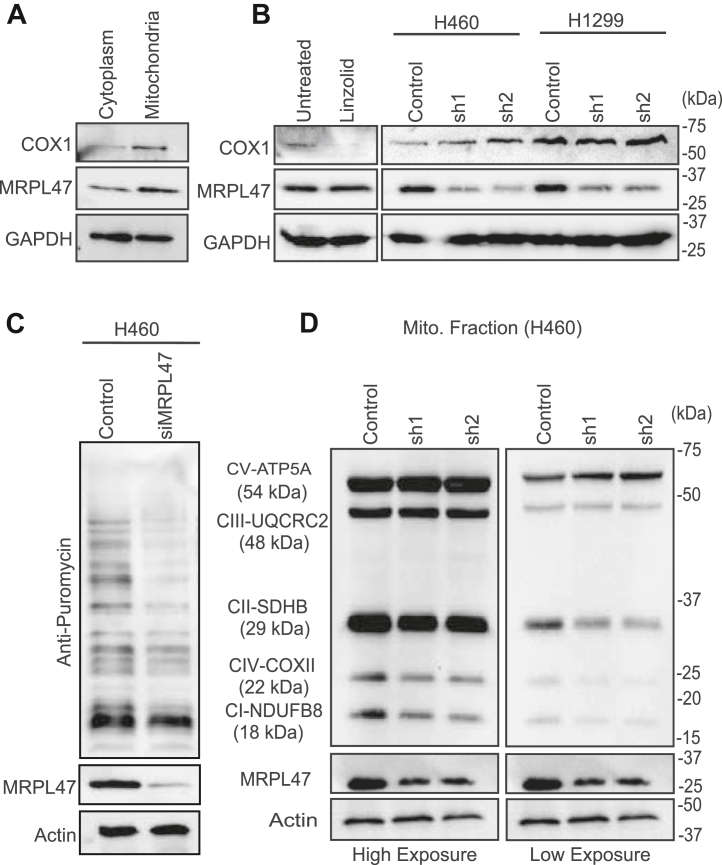
**Role of MRPL47 in Mitochondrial Function and Respiratory Complex Activity.***A,* Western blot analysis showing MRPL47 localization predominantly in the mitochondrial fraction, alongside COX1 (mitochondrial marker) and GAPDH (cytoplasmic marker) in H460 cells. *B,* COX1 protein levels in A549 and H1299 cells showing unchanged expression in control vs knockdown cells. A decrease in the COX1 level of linzolid treated cells indicate inhibition of mitochondrial translation. *C,* Surface SEnsing of Translation assay demonstrating a significant decrease in some but not all mitochondrial protein translation upon MRPL47 knockdown in H460 cells, confirming MRPL47's role in selective mitochondrial translation. *D,* Western blot analysis of mitochondrial respiratory complex subunits (Complexes I-V) in mitochondrial fractions from H460 cells. MRPL47 knockdown results in reduced levels of Complex I (NDUFB8), Complex II (SDHB), and Complex IV (COXII), while Complex III (UQCRC2) and Complex V (ATP5A) remain unaffected. For better appreciation of the mitochondrial protein expression, low exposure blots are shown with Actin and MRPL47 blots with same earlier exposure.

Previous studies have identified that knocking down MRPs such as MRPL12 and MRPS34, leads to a global reduction in the mitochondrial translation, highlighting their critical role in regulating mitochondrial protein synthesis ^25^. Based on these findings, we aimed to investigate the role of MRPL47 in mitochondrial translation beginning with an assessment of its localization. The Western blot results demonstrated the predominant localization of MRPL47 in the mitochondrial fraction of H460 cell line (Fig. 4*A*). Next, we examined the levels of COX1, a mitochondrially encoded protein in MRPL47 silenced cells. Surprisingly, MRPL47 knockdown did not affect COX1 expression, whereas a clear reduction was observed in the linezolid-treated samples confirming impaired mitochondrial translation (Fig. 4*B*). Motivated by these results, we sought to determine whether MRPL47 is required for the synthesis of global mitochondrial translation, or its role is limited to translation of specific mitochondrially-encoded proteins. To address this question, we performed a Surface SEnsing of Translation (SUnSET) assay for measuring global protein synthesis in MRPL47 silenced cells. This assay utilizes puromycin's ability to incorporate into the newly synthesized polypeptide chains, prematurely terminating translation. These puromycin labelled proteins can be further detected by using an anti-puromycin antibody. Our findings revealed a robust puromycin incorporation signal across multiple molecular weight bands, indicating an active global protein synthesis in the control cells. In contrast, MRPL47 knockdown cells exhibited a significant reduction in the puromycin signal of some proteins, but not all, validating our previous findings. (Fig. 4*C*). To further explore the involvement of MRPL47 in the functionality of OXPHOS system we utilized a total OXPHOS monoclonal antibody cocktail targeting multiple subunits of the respiratory chain complex crucial for oxidative phosphorylation. We observed a reduction in the Complex I (NDUFB8), II (SDHB), and IV (COXII) subunits while Complex III (UQCRC2) and Complex V (ATP5A) expression remained unchanged indicating selective impairment in the respiratory complexes, consistent with our previous findings. (Fig. 4*D*). This selective disruption within the electron transport chain subunits suggests specific dysfunctions. These findings suggest that MRPL47 silencing selectively impacts mitochondrial protein synthesis and the functionality of the OXPHOS system, thereby affecting cellular energy metabolism.

### Mitoribosomal dysfunction impairs OXPHOS further leading to ROS generation

**Figure 5:**
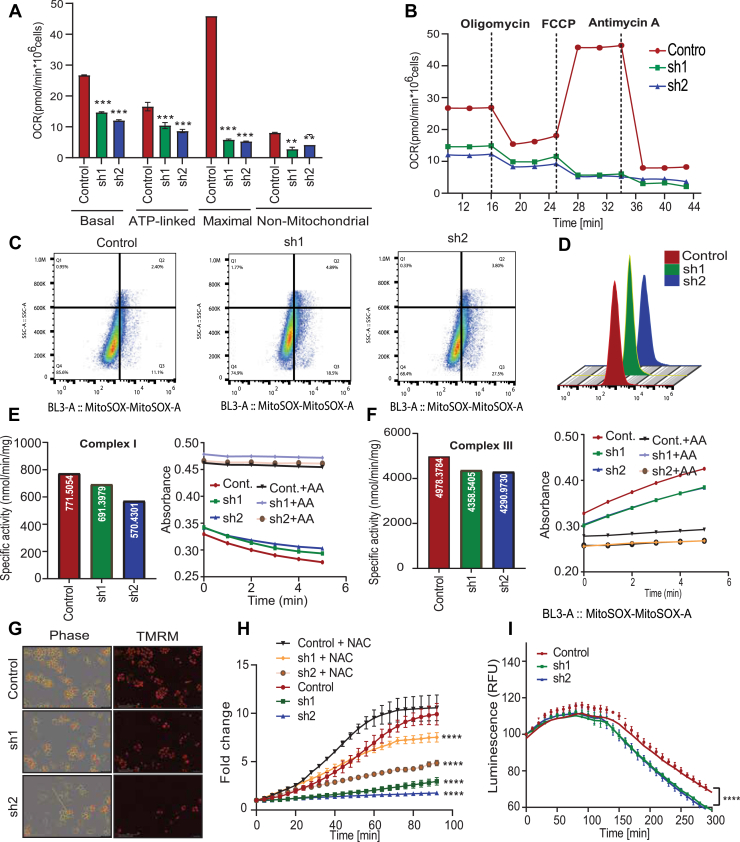
**Functional consequences of MRPL47 gene silencing on mitochondrial dynamics, oxidative stress, and cell growth.***A,* quantification of basal respiration, ATP-linked respiration, maximal respiration, and non-mitochondrial respiration based on OCR measurements. *B,* time-course representation of OCR changes in response to mitochondrial inhibitors in control and MRPL47 knockdown cells, demonstrating a substantial decrease in mitochondrial respiratory capacity in knockdown groups. *C,* MitoSOX-based flow cytometric detection of mitochondrial ROS production in control and MRPL47 deficient H460 cells Increased MitoSOX fluorescence corresponds to the higher levels of mitochondrial oxidative stress in MRPL47 knockdown cells. *D,* representative flow cytometry histogram overlays displaying MitoSox Red fluorescence intensity in control, sh1, and sh2 cells. MRPL47 knockdown cells exhibit a rightward shift, confirming elevated mitochondrial oxidative stress. *E and F,* enzymatic activity of mitochondrial Complex I and III measured *via* spectrophotometry. Specific activity (*left*) and absorbance over time (*right*) show a reduction in activity for both complexes in MRPL47 knockdown cells. Addition of specific inhibitors (Rotenone for Complex I, Antimycin A for Complex III) confirms specificity of the assays. *G,* incucyte images showing phase contrast and Tetramethylrhodamine methyl ester staining images of H460 cells to evaluate changes in mitochondrial membrane potential (ΔΨm). Loss of Tetramethylrhodamine methyl ester fluorescence signal in MRPL47 knockdown cells suggests depolarization of the mitochondrial membrane. *H,* rescue experiment validating ROS mediated inhibition of cell proliferation in MRPL47 silenced cells, with or without NAC treatment. Elevated ROS levels in sh1 and sh2 cells are rescued by NAC treatment. *I,* luminescence-based ATP assay showing a significant decline in ATP generation over time duration of 5 h in control and MRPL47 knockdown cells. A marked reduction in ATP production is observed in sh1 and sh2 groups, reflecting impaired mitochondrial function. NAC, N-acetyl-L-cysteine; OCR, oxygen consumption rates.

The biogenesis of Mitoribosome is crucial for regulation of mitochondrial respiration and cellular energy homeostasis. Often, mitoribosome dysfunction can contribute to impairment of the OXPHOS system which in turn leads to elevated ROS production causing detrimental effects on cellular function (21). To elucidate the function of MRPL47 in mitochondrial respiration, we utilized a florescent oxygen consumption rates (OCR) probe which is quenched by oxygen present in the medium. To investigate about the changes in oxygen consumption rate after knocking down MRPL47, we utilized a florescent OCR probe which is quenched by oxygen present in the medium. In this assay an oil layer is added on top of the assay medium to limit the oxygen diffusion. The results demonstrated that MRPL47 knockdown cells were showing that MRPL47 is necessary for optimal mitochondrial respiration (Fig. S3*B*). Further, we measured the OCR in MRPL47 silenced cells using high-resolution respirometry with the Oroboros O2K system. The oxygraph indicated a progressive decrease in the Baseline oxygen consumption over time in the MRPL47 silenced cells when compared to the control, while compound-specific injections (ce1-ce4) demonstrated differential effects on mitochondrial activity such as CE1 induced respiration, indicating diminished basal respiration in MRPL47-silenced cells. CE2 (Oligomycin) inhibited Complex I, hence validating the disruption of the respiratory chain. CE3 cyanide-p-trifluoromethoxyphenylhydrazone (FCCP), an uncoupler, elicited maximal electron flux, demonstrating an impaired oxidative phosphorylation due to the reduced response. CE4 (Antimycin A) completely obstructed respiration, emphasizing the dependency of mitochondria on OCR and the bioenergetic deficiencies resulting from MRPL47 ablation (Fig. S3*C*). Furthermore, OCR profiles displayed a significant reduction across basal, ATP-linked, maximal as well as nonmitochondrial respiration states strongly suggesting the critical role of MRPL47 in regulating mitochondrial respiration and OXPHOS activity (Fig. 5*A*). Upon sequential treatment with the inhibitors-oligomycin, FCCP, and antimycin A, control cells displayed a higher baseline and maximal respiration, while knockdown cells maintained consistently lower OCR, reinforcing mitochondrial dysfunction as a specific consequence of MRPL47 loss (Fig. 5*B*). The observed changes in OCR profiles, particularly reductions in ATP-linked and maximal respiration, suggest impaired electron transport chain (ETC) activity, leading to electron leakage and ROS generation. To validate this, flow cytometry analysis was performed using MitoSOX, a mitochondrial superoxide indicator showing elevated ROS levels in knockdown cells compared to the control (Fig. 5, *C* and *D*). Since, ETC complex I and III are considered major sites for ROS production, we aimed to perform a spectrophotometric assessment of enzymatic activities of these complexes. Complex I dysfunction, evidenced by reduced NADH oxidation, aligns with decreased NDUFB8 protein levels in MRPL47 knockdown cells, explaining the diminished OCR observed in the Oroboros O2k assay. Complex III dysfunction, despite unchanged UQCRC2 protein levels, reflects impaired enzymatic activity likely due to ETC destabilization from upstream defects in Complex I (Fig. 5, *E* and *F*). Furthermore, elevated ROS level disrupts the ETC, leading to a reduction in the proton gradient and mitochondrial membrane potential. Consistently, Tetramethylrhodamine methyl ester staining revealed a marked reduction in the fluorescence intensity of the knockdown cells, confirming impaired mitochondrial membrane potential and dysfunction upon MRPL47 silencing (Figs. 5*G* and S3*C*). According to our previous findings, silencing MRPL47 significantly reduced cell proliferation. To determine whether this effect was due to oxidative stress or ROS, we performed a cell proliferation assay by treating the cells with N-acetyl-L-cysteine (NAC), a well-known ROS scavenger. Treatment with NAC rescued the cell proliferation deficit**,** indicating the observed reduction in cell proliferation as a result elevated ROS levels (Fig. 5*H*). Since, impaired membrane potential also affects the proton gradient essential for ATP synthase activity, we further validated this through a luminescence assay showing significantly decreased ATP generation over a time period of 5 h in the knockdown groups (Fig. 5*I*). Collectively, these results demonstrate that MRPL47 plays a pivotal role in mitochondrial function by regulating respiration, oxidative stress, membrane potential, and energy production. Its silencing leads to mitochondrial dysfunction and impaired cellular proliferation, largely driven by increased ROS levels.

### Silencing of MRPL47 inhibits E2F pathway

**Figure 6:**
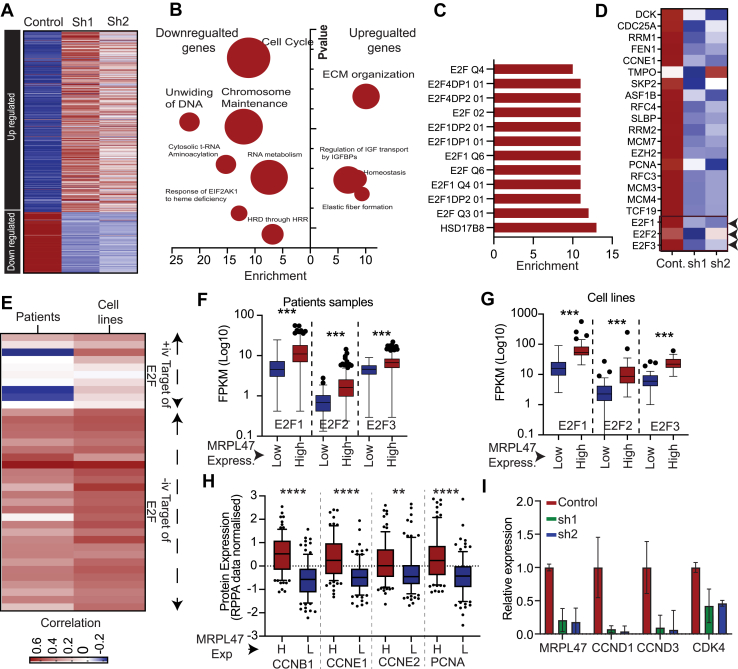
**Role of MRPL47 in E2F-Mediated Cell Cycle.***A,* differential Gene Expression Analysis showing upregulated/downregulated genes in MRL47 knockdown *versus* control groups. *B,* pathway Enrichment Analysis illustrates the major affected pathways and downregulation of Genes Involved in Cell Cycle and DNA Maintenance. *C,* enrichment of E2F targets as identified by metascape analysis. *D,* heatmap showing the expression of E2F target genes in control and MRPL47 downregulated samples as identified in RNA-sequencing. *E,* correlation of MRPL47 expression with expression of E2F target genes in TCGA-NSCLC patients and CCLE cell lines. The negative targets of E2F show negative correlation with MRPL47 and positive targets of E2F show positive correlation with MRPL47. *F and G,* Expression of various E2F genes in MRPL47 high and low expressing patients (*F*) and cell lines (*G*). *H,* protein expression (from TCGA RPPA data) of cell cycle regulators which are target of E2F in MRPL47 high and low groups. *I,* expression of CCND1, CCND3 and CDK4 was measured in control and MRPL47 knockdown samples and fold change relative to control are plotted. CCLE, cancer cell line Encyclopedia

To understand the molecular consequences of MRPL47 mediated cell growth inhibition, we performed RNA-seq analysis to identify differentially expressed genes in MRPL47-silenced cells compared to controls (Table S1). The heat map representation of differentially expressed genes indicated substantial changes in gene expression of MRPL47-silenced genes relative to the controls (Fig. 6*A*). Gene ontology enrichment analysis using down regulated genes highlighted enrichment of pathways related to cell cycle progression, chromosome maintenance and DNA unwinding, highlighting a functional link between MRPL47 and genomic integrity (Fig. 6*B*). Further, we identified that genes whose expression was decreased in MRPL47 knockdown cells were regulated by E2F (Fig. 6*C*). Further detailed analysis showed the downregulation of various positive E2F targets in MRPL47 silenced cells. Whereas many genes negative regulated by E2F, were downregulated in MRPL47 knockdown cells (Fig. 6, *D* and *E*). To corroborate these findings, we analyzed expression data from TCGA patient samples, demonstrating that key E2F genes (E2F1, E2F2, and E2F3) were highly expressed in samples with high MRPL47 expression (Fig. 6*F*). This observation was further corroborated using cell line data (Fig. 6*G* Furthermore, analysis of TCGA reverse phase protein array (RPPA) protein expression data revealed that established E2F target proteins, including CCNB1, CCNE1, CCNE2, and PCNA (22), exhibited higher expression levels in samples with high MRPL47 expression (Fig. 6*H*). Direct assessment of cyclin and CDK expression, known E2F targets, in our MRPL47 knockdown samples showed significantly reduced levels of these critical cell cycle regulators (Fig. 6*I*). Collectively, these comprehensive findings demonstrate that the depletion of MRPL47 profoundly impairs cell cycle regulation, largely through the intricate inhibition of the E2F pathway.

### MRPL47 regulates cellular senescence and the ROS-p38mitogen-activated protein kinase signaling axis

**Figure 7:**
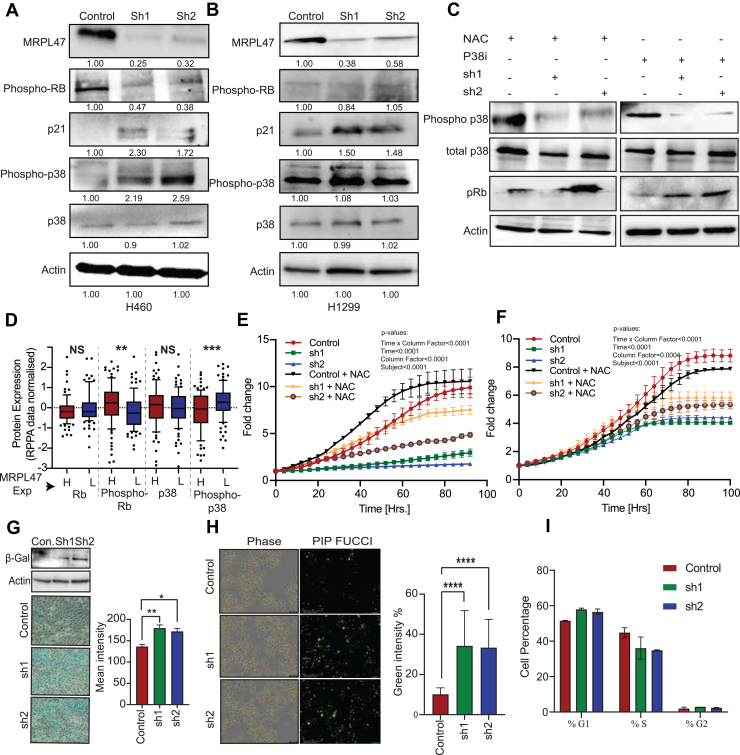
**Loss of MRPL47 drives cellular senescence and cell cycle arrest through p38MAPK-CDKN2A Pathway.***A and B,* Western blot showing the phosphorylation levels of key proteins including phospho-Rb, p21, phospho-p38 and total p38 in H1229 cells (*A*) and H460 cell (*B*). *C,* Western blot analysis showing the effects of p38 inhibitor (P38i) and antioxidant NAC treatment on p38 phosphorylation, and phospho-Rb levels in MRPL47 knockdown (sh1, sh2) and control H460 cells. *D,* protein level expression (RPPA data) of Rb, Phospho-Rb, p38 and phopsho-p38 in TCGA-NSCLC patients. *E and F,* the control or MRPL47 knockdown H460 (*E*) and H1299 (*F*) cells were treated with NAC/control and proliferation was measured using Incucyte. A two way ANOVA was performed to test the rescue of NAC on cell proliferation. Figure 7*E* is same as Figure 5*I* with different interpretation. *G,* beta-galactosidase staining to check senescence in MRPL47 knockdown H460 cells. The *blue* staining represents senescent cells. Western blot analysis of Beta-galactosidase in H460 cells, with quantification of staining intensity (*right*) showing increased senescence in knockdown cells compared to control. *H,* representative images of the PIP-FUCCI reporter assay showing G1 cell cycle arrest (*high green intensity*) in MRPL47 knockdown. Quantification of *high green intensity* cells is shown on the right. *I,* flow cytometry cell cycle analysis in MRPL47 knockdown. The Bar graph shows the percentage of cells in G1, S, and G2 phases. NAC, N-acetyl-L-cysteine; NSCLC, non-small cell lung cancer; TCGA, The Cancer Genome Atlas.

The tight regulation of E2F-mediated transcription by the Rb protein is a cornerstone of cell cycle control. Growth factor signaling triggers Cyclin-CDK complexes to phosphorylate Rb, liberating E2F to activate target gene expression. Investigating the mechanism behind E2F pathway inhibition in our model, we observed a significant reduction in the phosphorylated form of Rb in MRPL47 knockdown cells (Fig. 7, *A* and *B*). This suggested that MRPL47 depletion impairs Cyclin-CDK activity, leading to reduced Rb phosphorylation and subsequent lower E2F activity.

Considering that p21 is a potent inhibitor of CDK2-CyclinE, a key complex involved in Rb phosphorylation, we examined p21 expression. Indeed, MRPL47 knockdown significantly increased p21 levels, which corresponded with the decreased phospho-Rb (Fig. 7, *A* and *B*).

Given our previous finding that MRPL47 knockdown increases ROS, and the established link between ROS, p38 activation, and p21 upregulation (23, 24)**,** we explored this potential pathway. Analysis of p38 phosphorylation revealed increased phospho-p38 levels upon MRPL47 knockdown, indicating p38 activation (Fig. 7, *A* and *B*). To confirm that elevated ROS is central to this cascade, we treated MRPL47 knockdown cells with the ROS scavenger NAC. NAC treatment effectively rescued phospho-Rb levels and reduced p38 phosphorylation, demonstrating the critical role of ROS in this regulatory axis (Fig. 7*C*). Clinical relevance was assessed using TCGA RPPA data from NSCLC patients stratified by MRPL47 expression. Patients with high MRPL47 levels exhibited significantly higher Rb phosphorylation and lower p38 phosphorylation, with total protein levels unchanged (Fig. 7*D*). To determine if the increased ROS resulting from MRPL47 knockdown was directly responsible for the observed cell growth inhibition, control and MRPL47-knockdown H460 and H1299 cells were treated with the antioxidant NAC. NAC treatment significantly rescued the anti-proliferative effects induced by MRPL47 depletion, indicating that ROS accumulation plays a crucial role in mediating the growth inhibition caused by MRPL47 knockdown (Fig. 7, *E* and *F*). Given that increased p21 and decreased Rb phosphorylation are known triggers for cellular senescence and G1 cell cycle arrest (25), we investigated these phenotypes in MRPL47 knockdown cells. Beta-galactosidase staining, and Western blotting confirmed a significant increase in senescent cells (Fig. 7, *G* and *H*). Cell cycle analysis demonstrated a pronounced accumulation in the G1 phase as demonstrated by PIP-FUCCI live-cell imaging (Fig. 7*I*), further validated by cell cycle analysis using fluorescence-activated cell sorting (FACS) (Fig. 7*J*).

In summary, our findings delineate a critical regulatory pathway involving MRPL47, ROS, p38, and p21. Loss of MRPL47 elevates cellular ROS, activating the p38 pathway and driving the upregulation of p21. Increased p21 inhibits Cyclin-CDK activity, resulting in Rb hypophosphorylation and E2F inactivation, ultimately leading to cellular senescence and G1 phase arrest.

## Discussion

Mitoribosomes, protein complexes present inside mitochondria plays a piovotal role in translating mitochondrial mRNA into proteins essential vital for oxidative phosphorylation (OXPHOS). Dysregulation of these mitochondrial ribosomal proteins (MRPs) has been associated with cancer progression through alteration in mitochondrial metabolism, impaired oxidative phosphorylation and increased production of ROS underscoring their therapeutic potential (5). We identified MRPL47 a recurrently amplified and overexpressed gene in NSCLC. Our study delineates the crucial role of MRPL47 in NSCLC tumor progression by regulating mitochondrial function. Loss of MRPL47 compromised mitochondrial translation resulting in decreased expression of selected proteins of Complex I, II and IV subunits of OXPHOS system, thus affecting ETC function and consequently increasing ROS levels. Such malfunction of ETC further lead to altered mitochondrial membrane potential, decreased ATP generation and oxygen consumption rate in the MRPL47 silenced cells. Mechanistically, our findings highlight the link between MRPL47 and mitochondrial dynamics *via* activation of the p38 mitogen-activated protein kinase (MAPK)-Rb-E2F pathway resulting in G1 arrest and cellular senescence. These findings underscore MRPL47's significance in mitochondrial homeostasis and its potential as a therapeutic target in Lung cancer.

MRPL47 uniquely influences mitochondrial protein synthesis by selectively regulating translation of ETC subunits of complex I (NDUFB8), II (SDHB) and IV (COX II), while not showing any relation with other proteins of subunits III (UQCRC2) and V (ATP5A). This observation supports the recent understanding that all the mitoribosome are not same (26). This selective regulation of mitochondrial translation by MRPL47 was further confirmed by SUnSET assay, demonstrating that silencing MRPL47 doesn't impact the synthesis of all mitochondrial-encoded proteins. Interestingly, the reduced protein expression of ETC complexes I and III subunits was accompanied with a considerable decrease in their enzymatic activity, highlighting the functional implications of MRPL47 silencing. Since, these complexes are also major contributors of ROS production, their dysfunction likely contributes as a mechanistic basis for the observed increased in ROS levels.

Although our findings suggest that MRPL47 may not be directly required for the translation of Complex III subunit protein, its involvement in Complex I translation may indirectly influence Complex III activity. Moreover, the decrease in protein expression of Complex IV subunit (COX II) may be a consequential outcome of compromised mitochondrial translation and malfunction. Furthermore, increased ROS production may also destabilize Complex IV indicating a widespread change in mitochondrial translation and homeostasis, compromising electron transport chain function and mitochondrial efficiency.

Our findings elucidate the molecular factors influencing the dependency of cancer cells on MRPL47. We comprehensively investigated to demonstrate a strong correlation between dependency on MRPL47 and expression of the tumor suppressor p21, a well-established regulator of cell cycle progression and senescence (27). Consequently, an increased p21 protein expression was observed in MRPL47 silenced cells exhibiting growth arrest which was in line with our previous findings. This upregulation was associated with increased phosphorylation of Rb and a decrease in cyclins and CDKs levels, leading to G1 cell cycle arrest. These results correspond with earlier studies connecting p21 to the ROS-p38 MAPK pathway in cellular senescence (28) and mitochondrial dysfunction.

A critical aspect of our study is to understand how MRPL47 play role in regulating ROS signalling. The increased level of ROS levels in MRPL47 silenced cells were found to be associated with altered mitochondrial membrane potential (ΔΨM), lower oxygen consumption rates and reduced ATP synthesis collectively signifying mitochondrial dysfunction. Furthermore, to understand whether ROS is mediating the cell growth inhibition in the silenced cells, NAC treatment was provided to scavenge ROS. The cell growth was rescued, confirming that ROS mediates the phenotypic consequences of MRPL47 knockdown. The downstream effects of elevated ROS were notably apparent in the activation of the p38 pathway, a key modulator of cellular stress responses and carcinogenesis (29). The phosphorylation of p38 and its downstream target, p21, indicates the role of ROS-p38-MAPK signalling in the regulation of cell cycle arrest. Additionally, RNA sequencing analysis confirmed that MRPL47 inhibits cell growth by downregulating E2F, a key regulator of cell cycle, showing consistency with our findings. These findings highlight the association between MRPL47, ROS signalling, and cell cycle regulation.

The therapeutic significance of MRPL47 was highlighted by its amplification and overexpression in several cancer types, especially NSCLC. Analysis of TCGA data demonstrated that MRPL47 amplification is substantially correlated with its expression, aligning with prior research indicating that gene amplification serves as a catalyst for oncogene overexpression (30). In NSCLC patients, elevated MRPL47 expression and increased copy number correlated with poor prognosis, reinforcing its potential as a prognostic biomarker.

Our findings illustrate that Mitoribosomes are not universal, and it seems their composition changes depending upon the target mRNA. Subsequent research is required to delineate the domain of MRPL47's substrate's specificity through methods like cryo-electron microscopy which will provide an understanding of its unusual form of its distinct translational control. *In vivo* investigations are crucial to confirm the therapeutic efficacy of targeting MRPL47 in cancer models. Given the relationship between mitochondrial dysfunction and the tumor environment, MRPL47 mediated ROS generation's effect on immune cell recruitment and activation is crucial to investigate.

## Experimental procedures

### Cell culture and reagents

Human derived cell lines H1299 cells were obtained from American type culture collection. H460, A549 and AGS were obtained from National Centre for Cell Sciences (NCCS). The autheticity of cell was confirmed using STR profiling. H1299 and H460 cell lines were cultured in RPMI medium whereas, A549 and AGS cell lines were cultured in F-12 K medium supplemented with 10% fetal bovine serum, 100 U/ml Penicillin & 100 μl/ml Streptomycin (Gibco). Cell lines were maintained in a CO2 incubator at 37 °C in a 5% CO_2_ humidified atmosphere.

### Generation of stable knockdown cells

The MRPL47 gene was silenced in LUAD cell lines with the application of siRNA or lentiviral-mediated expression of shRNA. Western blot analysis and semi-quantitative PCR/qPCR were conducted to assess the efficiency of MRPL47 silencing. For shRNA-mediated knockdown, two distinct shRNAs were employed for MRPL47 silencing: sh1 CTTGCCTTATGTGGACCATTT and sh2 GTAGATTCCATGGATGCATTA. pLKO.1 puro, a third generation lentiviral plasmid, for the cloning and silencing of MRPL47 expression in LUAD cell lines was acquired from Addgene (Plasmid #8453). This was accomplished by designing a 21 bp target sequence for MRPL47 and subsequently cloning it into the Age1-EcoR1 sites of the pLKO vector. The Lenti-X 293T cell line was utilized for the generation of viral supernatants and subsequent infections. Transfection was performed using Xfect polymer (TaKaRa Bio, cat.#631317) in accordance with the manufacturer's guidelines. The transfected cells were selected with 2.5 μg/ml puromycin (Sigma, cat.#P8833) for at least 48 h. For siRNA mediated RNA interference, cells were transfected with 5 nM Ambion Silencer Negative Control #1 siRNA (Thermo Fisher Scientific, cat #AM4611) and MRPL47 siRNA (siMRPL47) utilizing Lipofectamine RNAiMAX transfection reagent (Thermo Fisher Scientific, cat #13778100), in accordance with the manufacturer's guidelines. The employed target sequence was as follows: siMRPL47 GACAUCUUUGGAAGAAUCA. Finally, these stable knockdown cells were harvested for protein and RNA extraction.

### Semi-quantitative/Quantitative real-time PCR

Total RNA was extracted utilizing TRIzol reagent (Ambion, cat.#15596018), and 500 ng of total RNA was employed to synthesize cDNA with iScript RT mix (Biorad, cat.#1708840), according to the manufacturer's instructions followed by a semi-quantitative PCR or a quantitative real time PCR employing Emerald GT PCR mix (TaKaRa, cat.#RR310A) and universal SYBR Green mix (Biorad, cat#1725271) in a thermal cycler.The ΔCt method was employed to quantify the mRNA levels of the target gene, normalized against TPT1, housekeeping gene. The 2^−ΔΔCt^ technique was employed to compare the mRNA levels of each target gene, and the relative amplification values were illustrated in the graph. The following primer sequences were used for MRPL47_FW: 5′-TGGAAGAATCATCTGGCACA- 3′; MRPL47_RV: 5′-ACTTTTGGGCTTCAGCAAGA-3′ TPT1_FW: 5′-GATCGCGGACGGGTTGT-3′ TPT1_RV: 5′-TTCAGCGGAGGCATTTCC-3′

### Cell proliferation assay

To assess cell viability and proliferation rate post-knockdown, we conducted an MTT tassay utilizing yellow tetrazolium MTT [3-(4,5-dimethylthiazol-2-yl)-2,5-diphenyl-tetrazolium bromide] (Sigma, cat. #475989). 20,000 cells per well were seeded in a 96-well plate and incubated with MTT for 4 h in a CO2 incubator followed by the addition of DMSO to solubilize the purple formazan complex, thereafter, measuring the absorbance at 570 nm with a spectrophotometer. For each cell type, the linear correlation between cell counts and signal output was determined, enabling the quantification of variations in the rate of cell proliferation.

### Wound healing assay

The migration of cells was assessed using a scratch wound assay performed on the Incucyte Live-Cell Analysis System (Sartorius) equipped with the Scratch Wound module. Cells were seeded into an Incucyte imagelock 96-well plate (Sartorius, cat. #BA-04856) at a density of 50,000 cells/well. Subsequently, uniform scratches were created in the cell monolayer using the Incucyte WoundMaker tool. Images of the wound area were automatically captured at an interval of 4 h over a period of 24 h using the Incucyte system. The wound width and confluence were quantified using the Incucyte Scratch Wound module, which calculates cell migration based on the decrease in the wound area over time. Data were analyzed using Incucyte software (https://www.sartorius.com/en/products/cell-analysis/incucyte-live-cell-analysis-system) and are presented as a percentage of wound closure relative to baseline. Finally, the wound healing % *versus* Hour's graph was plotted using Graphpad Prism software (https://www.graphpad.com/scientific-software/prism/).

### Colony suppression assay

To identify the ability of knockdown cells to grow into colonies, we seeded 500 cells/well in a 12-well plate and allowed them to grow for 2 weeks. The media was replaced every 4 to 5 days for 12 days followed by staining the colonies using 0.25% crystal violet solution. Finally, the images were captured and analyzed.

### Western blot analysis

To identify the changes in protein expression of the knockdown cells, Western blot was performed using MRPL47 antibody. For protein isolation, cells were trypsinized and washed with cold PBS followed by addition of IP lysis buffer (Pierce #87787) and protease inhibitor cocktail (Halt protease inhibitor cocktail #87786) to the pellet. The cell lysates were centrifuged at 13,000*g* for 15 min and supernatants were collected. The total protein concentration was estimated before loading onto polyacrylamide gel by BCA protein assay kit (Pierce #23227) and a total of 50 μg of cell lysate was loaded and resolved in 12% polyacrylamide gel (Biorad TGX FastCast Acrylamide Kit 12% 161-0175)). The gel was blotted in polyvinylidene difluoride (PVDF) membrane (Immun-Blot PVDF Membranes for Protein Blotting, Biorad #1620177) and the membrane was blocked using a 5% blocker solution (Blotting Grade Blocker, Biorad # 1706404). The membrane was incubated with MRPL47 (Immunotag #ITT03673) and internal control Actin B (ABclonal #AC004) or GAPDH (ABclonal #AC002) antibodies were used. The membrane was later incubated with HRP-tagged goat anti-mouse (Bio Rad #172-1011) or anti-rabbit (Invitrogen #31460) secondary antibodies. All the steps were followed by washing with TBS (Bio Rad #1706435) comprising 0.1% Tween 20 (Polysorbate 20, MP Biomedicals #103168). The bound antibody complexes were visualized using ECL Western Blotting substrate (Pierce #32209) or femtoLUCENT PLUS HRP chemiluminescent reagents (G Biosciences #786-003).

### Determination of mitochondrial ROS production

The intracellular mitochondrial ROS level was determined using MitoSOX red mitochondrial superoxide indicator (Invitrogen #M36008). 0.3 × 10^6^ cells were seeded in a six well plate and kept for growing inside the CO2 incubator overnight. Next day cells were treated with 2 mM MitoSOX, mitochondrial superoxide indicator for 10 min in a CO2 incubator at 37 °C & 5% CO2. The treated samples were washed three times with PBS. The cells were then trypsinized and collected in FACS tubes. Unstained & unstimulated cells were used as a control while performing FACS in atteuneNXT Flow cytometer (Thermo Fisher Scientific). Finally, the fluorescence was recorded at 510/580 nm and the results were analyzed in flow cytometry standard express software.

### Senescence assay

To identify the senescence in MRPL47 knockdown cells, Beta-galactosidase staining kit (CST #9860) was used. Control and experimental cells were allowed to continuously grow in a six well plate for the experiment for 1 week prior to the experiment to achieve senescence. Cells were then fixed with 1× fixating solution for 10 to 15 min at RT. Next, PBS wash was performed two times followed by addition of Beta galactosidase staining solution. The plate was then sealed tightly with a parafilm and incubated in a dry CO2 free incubator overnight. Next day the image was acquired using an optical microscope.

### Extracellular oxygen consumption assay

Changes in OCR after knocking down MRPL47 in H460 cells was measured using Oroboros O2k high-resolution respirometer (Oroboros Instruments). To evaluate OCR, 1 × 106 cells were resuspended in media and added to pre-calibrated chambers under constant stirring. Afterward, a standard substrate-uncoupler-inhibition titration protocol was followed. First, a basal respiration was recorded and then sequential injections of Oligomycin (Cayman #11341), FCCP (Cayman #15218) and Antimycin A (CST #33357) was used to inhibit complexes I and III respectively. Finally, OCR values were recorded in real time using DatLab software, normalized to cell count.

### SuNSET assay

To detect the changes in the newly synthesized protein synthesis, SUnSET assay was performed that measures the puromycin incorporated into nascent polypeptide peptide chains using anti-puromycin antibody. After silencing MRPL47, the cells were treated with 1 μM Puromycin (Sigma #P8833) for 30 min and incubated at 37 °C. Post treatment, the mitochondria were isolated using manufacturer's protocol using a mitochondrial isolation kit (Abclonal #Ab110170). Furthermore, 50 μg of mitochondrial extract was resolved using a 12% polyacrylamide gel. Following this the samples were transferred to a PVDF membrane (G Biosciences #GS-PVDF-302) and subsequently incubated in TBST buffer consisting of 5% Quick Blocker protein powder (G Biosciences #786–011) for 2 h at room temperature. Next, PVDF membrane was cut and incubated overnight at 4 °C with anti-Puromycin (DSHB #PMY-2A4), MRPL47 (Immunotag #ITT03673), GAPDH (ABclonal #AC002) antibodies. Following day, membranes were incubated for 1 Hour at room temperature in TBST buffer containing HRP tagged goat anti-mouse (Bio Rad #172–1011) or anti-rabbit (Invitrogen #31,460) secondary antibodies. All the membrane washing steps were performed four times at an interval of 10 min using TBS (HIMEDIA #ML029) along with 0.1% Polysorbate 20 (MP Biomedicals #103,168). Finally, to develop the image of the blot containing protein of interest, chemiluminescence detection method was performed using an ECL Western Blotting substrate (Pierce #32,209) or femto LUCENT PLUSHRP (G Biosciences #786–003) reagents.

### Mitochondrial Complex activity assay

The enzymatic activities of Complex I and III in isolated mitochondria was assessed using a single-wavelength spectrophotometric assay. Complex I activity was measured by monitoring NADH oxidation at 340 nm whereas, Complex III activity was assessed by following the reduction of cytochrome c at 550 nm. All the assays were performed at 30 °C and results were normalized to total protein.

### *In vivo* tumorigenesis

All animal procedures were performed in accordance with institutional guidelines and with approval from the Institutional Animal Ethics Committee of IIT Dharwad (Protocol # BSBE/05/2025). Six-week-old male NOD-SCID (NOD.Cg-Prkdcscid/J) mice were housed under pathogen-free conditions. For tumor xenograft establishment, 1 × 10^6^ viable H460 cells (control shRNA or shMRPL47) suspended in 100 μl sterile PBS were injected subcutaneously into the right flank of each mouse (n = 5 per group). Tumor growth was monitored beginning Day 9 post-injection and subsequently every alternate day until Day 17. Tumor length (L) and width (W) were measured using digital Vernier calipers. Tumor volume was calculated using the standard formula:

$$\text{Tumor}\;\text{Volume}\;\left({\text{mm}}^{3}\right)=0.5\times \text{Length}\times \left({\text{Width}}^{2}\right)\;{mm}^{3}$$

Body weight was recorded throughout the study to assess systemic toxicity. Mice were sacrificed by CO_2_ asphyxiation as per IAEC guidelines at the study endpoint. Tumor weight and volumes were measured after harvesting the tumors from the mice, and photographs were taken.

### Statistical analysis

Tumor volume data were analyzed using linear mixed effects models with log-transformed volumes, accounting for repeated measurements within individual mice. Models included fixed effects for treatment, time, and their interaction, with random intercepts for each mouse. Model selection was performed using Akaike Information Criterion. Post-hoc comparisons were conducted using estimated marginal means with Tukey adjustment for multiple comparisons. Statistical analysis was performed in R (version 4.5.0) using the lme4 and lmerTest packages, following best practices for xenograft tumor growth analysis (31).

### Western blot analysis

Cells were lysed in ice-cold RIPA buffer (G-Biosciences #786-489) supplemented with protease inhibitor cocktail (Halt protease inhibitor cocktail #87786). Protein concentration was determined using BCA Assay (Thermo Fisher Scientific Pierce #23227). Equal amounts of protein 50 μg per sample were resolved using 12% SDS-PAGE gels and transferred to PVDF membrane (Biorad #1620177). Further, membrane were blocked in 5% BSA solution (Blotting Grade Blocker, Biorad # 1706404) and incubated overnight at 4 °C with primary antibodies- MRPL47 (Immunotag #ITT03673), cleaved PARP1 (proteintech #60555-1-Ig), Caspase 3 (proteintech #19677-1-AP) and internal control β-actin (ABclonal #AC004). The specificity of each primary antibody was verified in accordance with manufacturer's datasheet and previously published literature. For phospho-specific antibodies, specificity was determined by showing the presence of band at the expected molecular weight. For MRPL47 and other gene-targeted proteins, loss of band in shRNA knockdown down cell lines was used as an internal validation of antibody specificity. After incubation with HRP-conjugated secondary antibodies anti-mouse (Bio Rad #172-1011) or anti-rabbit (Invitrogen #31460). Proteins were visualized using ECL Western Blotting substrate (Pierce #32209) or femtoLUCENT PLUS HRP chemiluminescent reagents (G Biosciences #786-003) and imaged on Chemidoc (Bio-Rad). Band intensities were quantified using GelAnalyzer 23.1 software.

Antibody specificity was validated through multiple independent approaches. Primary antibodies included anti-MRPL47 anti-β-actin anti-phospho-Rb, anti-p21, anti-phospho-p38 MAPK (Thr180/Tyr182), and anti-p38 MAPK. Western blot analysis confirmed antibody specificity, showing single bands at expected molecular weights. MRPL47 antibody specificity was validated genetically by demonstrating reduced signal in cells transfected with multiple independent MRPL47-targeting shRNAs compared to non-targeting control. Phospho-specific antibodies (anti-phospho-Rb and anti-phospho-p38) were validated pharmacologically by demonstrating signal reduction upon treatment with specific pathway inhibitors (CDK4/6 inhibitor for phospho-Rb; p38 MAPK inhibitor for phospho-p38), while total protein levels remained unchanged. All experiments included appropriate positive controls (cell lysates with known target expression) and negative controls (primary antibody omission showing no signal). All antibodies were obtained from commercial sources and have been extensively validated by manufacturers and in peer-reviewed publications.

### Cell line authentication

H460 and A549 cells were procured from the National Centre for Cell Science (NCCS), Pune, India. H1299 cells were procured from American type culture collection. The authenticity of cells was confirmed using Short Tandem Repeat profiling. Cell line was tested and confirmed to be Mycoplasma-free by PCR-based detection. To maintain authenticity, only low-passage cultures were used for experiments, and cells were routinely examined for morphology, doubling time, and growth behavior to ensure consistency with the known characteristics.

## Data availability

All the data is available as supplementary file.

## Supporting information

This article contains supporting information.

## Conflict of interest

The authors declare that they have no conflicts of interest with the contents of this article.

## Supporting information

Supplementary figures

Expression data

**Abbreviations**
ETC, electron transport chain
MAPK, mitogen-activated protein kinase
MRP, mitoribosomal protein
NAC, N-acetyl-L-cysteine
NSCLC, non-small cell lung cancer
OCR, oxygen consumption rates
ROS, reactive oxygen species
TCGA, The Cancer Genome Atlas
