# Supplementary material for: MRPL47 deficiency drives mitochondrial dysfunction via ROS-p38-p21 signaling in non-small cell lung cancer
Source: J Biol Chem. 2025 Dec 15;302(2):111058. doi: 10.1016/j.jbc.2025.111058 (PMC12809090; doi:10.1016/j.jbc.2025.111058)
Supplement: Supplementary figures [file mmc1.pdf]

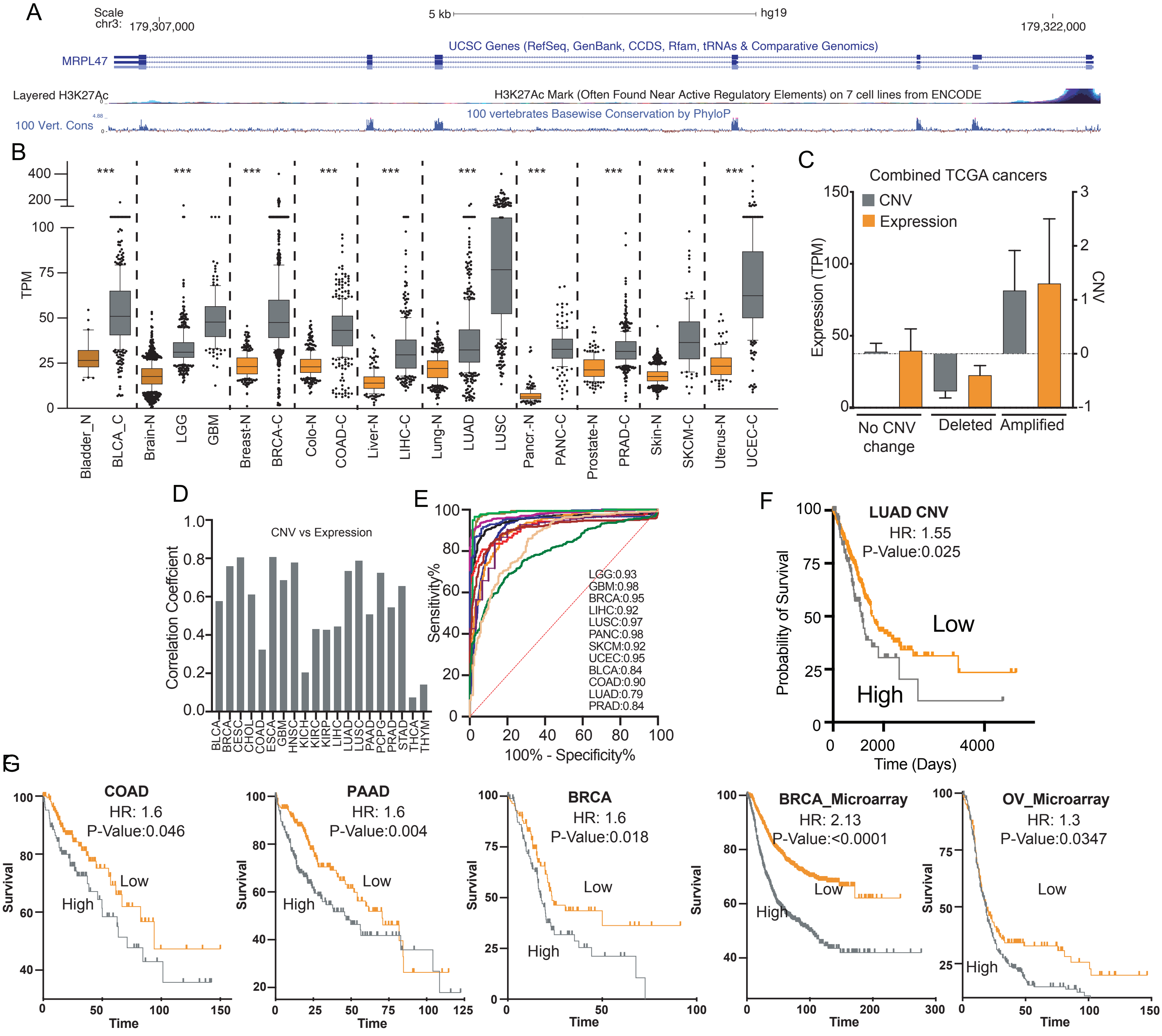

Supplementary Figure1: **A)** Structure of MRPL47 gene. Chip-seq data showing enrichemnt of H3K27Ac. **B**) Expression of MRPL47 RNA across multiple common cancer and correponding normal samples. **C**) Bar chart showing the CNV and expression association of MRPL47 gene in all TCGA cancer types. **D**) Correlation between expression and CNV was calculated and plotted to show the high association of MRPL47 expression and CNV. **E**) ROC plots showing the cancer specific expression of MRPL47. F) Prognostic association of MRPL47 CNV in LUAD **G**) Prognostic role of MRPL47 expression in given cancer types.

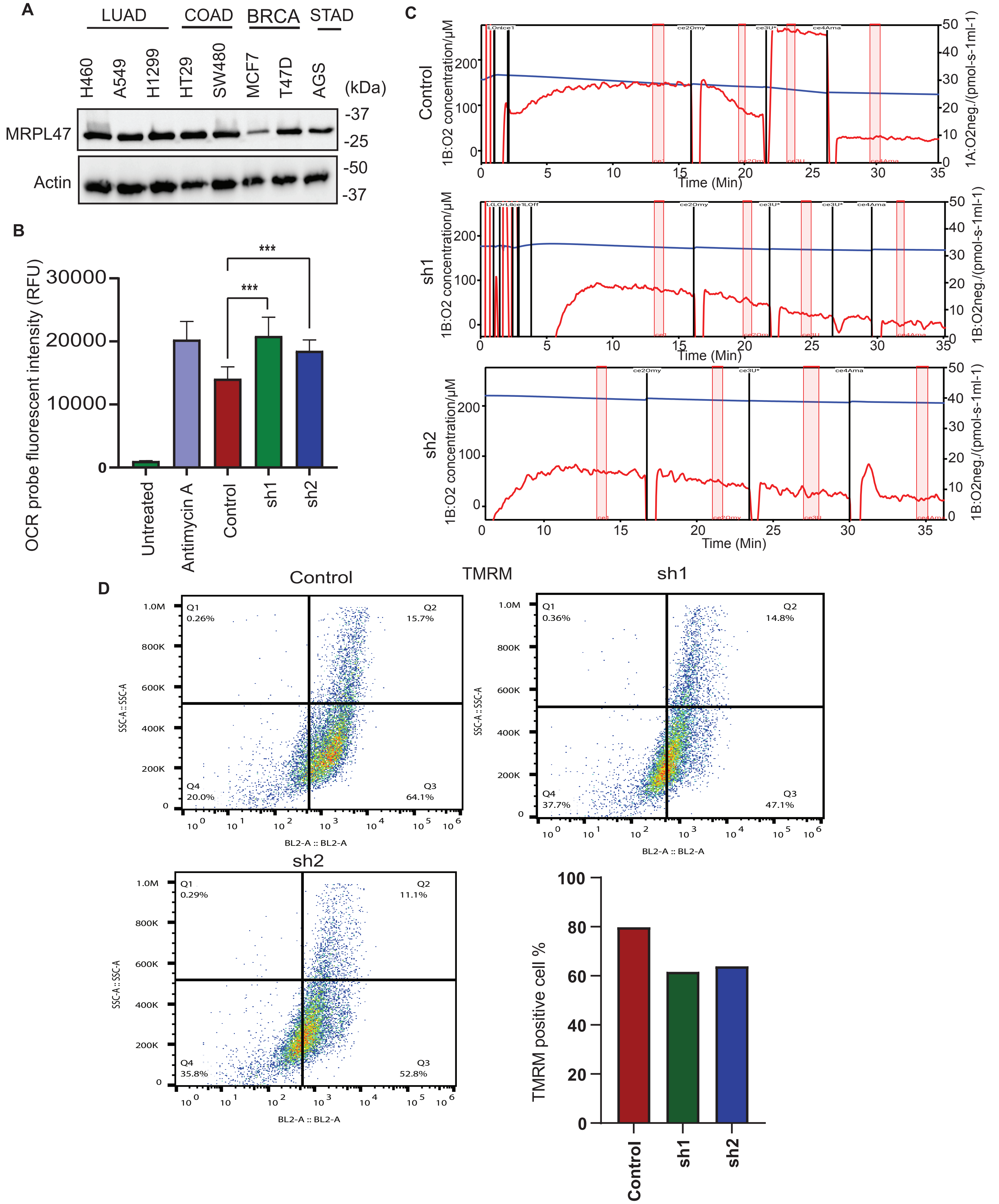

Supplementary Figure 3: **A)** Western blot demonstratingprotein expression of MRPL47 across various cancer cell lines. **B)**. Bar plot showing relative fluorescence intensity of OCR probe in MRPL47 knockdown H460 cells. Antimycin A serves as a positive control. ***p<0.001. **C).** Oroboros high resolution respirometry OCR traces in control and MRPL47 knockdown H460 cells. **D)** TMRM flow cytometry plots and quantification showing deceased mitochondrial membrane potential in MRPL47 knockdown cells

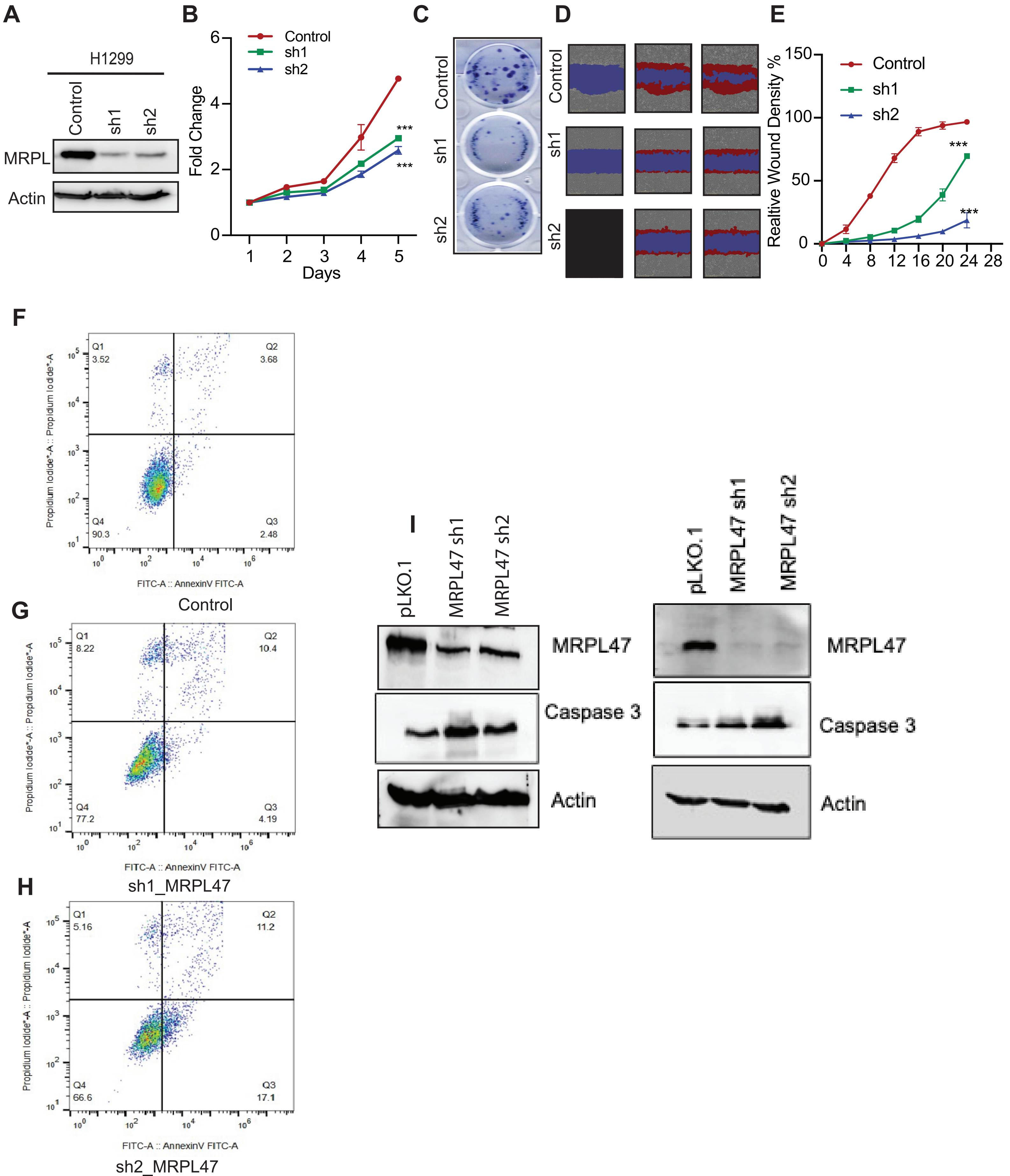

Supplementary Figure 2: MRPL47 knockdown suppresses cell proliferation, colony formation, and migration in LUAD cell lines H1299. (**A**) Western blot analysis confirming MRPL47 knockdown using two independent shRNAs. (**B**) Cell proliferation assay demonstrating reduced proliferation in MRPL47 silenced cells compared to control in H1299 over a given period. (**C**) Colony formation assay showing decreased colony-forming ability in MRPL47 knockdown cells in H1299. (**D**) Representative images of Scratch wound-healing assay displaying delayed wound closure in MRPL47 silenced cells compared to controls H1299 (**E**) Quantification of wound-healing assay indicating significantly reduced migration capacity in MRPL47 depleted cells H1299 cells over a time period of 24 hours. Statistical significance: (***denotes $p < 0.001$; ****denotes $p < 0.0001$). Error bars represent mean ± SD from triplicate experiments. (**F, G and H**). Control and MRPL47 kncokdown cells were stained with FITC-Annexin V and PI and subjected to Flow analyser. The increase in Apototic cells are visible in Q2 and Q3. (**I**) Control and MRPL47 kncokdown cells were lysed and western blot blot analysis was done to check the expression of MRPL47, Caspase 3 and Actin (**Same MRPL47 and Actin blots are used in figure 3A**).
